# From Classroom to Connection; Experiential Learning Disability Education in Psychiatry Training

**DOI:** 10.1192/bjo.2026.11305

**Published:** 2026-06-30

**Authors:** Hayley Lawrence

**Affiliations:** DHCFT, Derbyshire, United Kingdom

## Abstract

**Aims:**

People with learning disabilities experience significant health inequalities, yetundergraduate medical education often provides limited opportunities for sustained,meaningful engagement with this population outside clinical settings. This cancontribute to anxiety, uncertainty, and communication barriers for future doctors. Thecollaboration between Transition2 (T2), a specialist education provision for youngadults with special educational needs and disabilities (SEND), and the PsychiatryTeaching Unit (PTU) at Derbyshire Healthcare NHS Foundation Trust was developedto address this gap. The projectaimed to embed experiential, learning disabilityfocused teaching within undergraduate psychiatry placements, prioritising livedexperience, communication, and relational learning.

**Methods:**

During undergraduate psychiatry placements, medical students spent structured timeat Transition2, engaging with young adults with learning disabilities throughfacilitated sessions and informal interactions. Activities included shared discussions,collaborative tasks, and reflective conversations, designed to support authenticengagement rather than observational learning. Teaching was informed byexperiential education principles, including Kolb’s experiential learning cycle,enabling students to experience, reflect, conceptualise, and apply learning related toholistic, person-centred care. Sessions were co-designed with Transition2 staff toensure they provided a safe, respectful, and empowering environment for learners.

Evaluation was qualitative, drawing on feedback from students, staff, and Transition 2 participants.

**Results:**

Feedback from students and staff demonstrated improved communication skills,increased confidence, and deeper understanding of learning disability within a real-world context. Students valued the opportunity to engage openly with individuals withlived experience, describing learning that extended beyond traditional classroom orclinical teaching. Many reflected on feeling genuinely invested in learners’ journeysand reported enhanced empathy and awareness of how language, pace, andenvironment influence communication. Transition2 learners benefited fromopportunities to practise communication, express personal perspectives, and interactwith future clinicians in a collaborative, non-clinical setting. The partnershipaddressed a critical gap in medical education by enablingrelational learning rarelyachievable in standard placements and successfully translated experiential learning theory into meaningful practice.

**Conclusion:**

The Transition 2 and PTU partnership demonstrates an innovative, exciting model for learning disability education within undergraduate psychiatry. By embedding livedexperience and fostering respectful, reciprocal relationships, the programme enhances communication skills, empathy, and confidence among future doctorswhile empowering young adults with learning disabilities. Similar partnerships couldbe embedded within community education settings, colleges, and supportedemployment services, contributing to a more inclusive, informed, and compassionatehealthcare workforce.

